# Characterization and profiling of immunomodulatory genes of equine mesenchymal stromal cells from non-invasive sources

**DOI:** 10.1186/scrt395

**Published:** 2014-01-13

**Authors:** Catharina De Schauwer, Karen Goossens, Sofie Piepers, Maarten K Hoogewijs, Jan LJ Govaere, Katrien Smits, Evelyne Meyer, Ann Van Soom, Gerlinde R Van de Walle

**Affiliations:** 1 Department of Reproduction, Obstetrics and Herd Health, Ghent University, Salisburylaan 133, 9820 Merelbeke, Belgium; 2 Department of Nutrition, Genetics and Ethology, Ghent University, Salisburylaan 133, 9820 Merelbeke, Belgium; 3 Laboratory of Biochemistry, Faculty of Veterinary Medicine, Ghent University, Salisburylaan 133, 9820 Merelbeke, Belgium; 4 Baker Institute for Animal Health, College of Veterinary Medicine, Cornell University, Hungerford Hill Road, Ithaca, NY 14853, USA

## Abstract

**Introduction:**

Mesenchymal stromal cells (MSCs) have been extensively studied for their promising capabilities in regenerative medicine. Although bone marrow is the best-known source for isolating equine MSCs, non-invasive alternative sources such as umbilical cord blood (UCB), umbilical cord matrix (UCM), and peripheral blood (PB) have also been reported.

**Methods:**

Equine MSCs from three non-invasive alternative sources were isolated from six individual mares (PB) and their foals (UCB and UCM) at parturition. To minimize inter-horse variability, the samples from the three sources were matched within the same mare and for UCB and UCM even within the same foal from that specific mare. The following parameters were analyzed: (i) success rate of isolation, (ii) proliferation capacity, (iii) tri-lineage differentiation ability, (iv) immunophenotypical protein, and (v) immunomodulatory mRNA profiles. Linear regression models were fit to determine the association between the source of MSCs (UCB, UCM, PB) and (i) the moment of first observation, (ii) the moment of first passage, (iii) cell proliferation data, (iv) the expression of markers related to cell immunogenicity, and (v) the mRNA profile of immunomodulatory factors, except for hepatocyte growth factor (HGF) as no normal distribution could be obtained for the latter variable. To evaluate the association between the source of MSCs and the mRNA expression of HGF, the non-parametric Kruskal-Wallis test was performed instead.

**Results:**

While equine MSCs could be isolated from all the UCB and PB samples, isolation from UCM was successful in only two samples because of contamination issues. Proliferation data showed that equine MSCs from all three sources could be easily expanded, although UCB-derived MSCs appeared significantly faster in culture than PB- or UCM-derived MSCs. Equine MSCs from both UCB and PB could be differentiated toward the osteo-, chondro-, and adipogenic lineage, in contrast to UCM-derived MSCs in which only chondro- and adipogenic differentiation could be confirmed. Regardless of the source, equine MSCs expressed the immunomodulatory genes CD40, CD80, HGF, and transforming growth factor-beta (TGFβ). In contrast, no mRNA expression was found for CD86, indoleamine 2,3-dioxygenase (IDO), and tumor necrosis factor-alpha (TNFα).

**Conclusions:**

Whereas UCM seems less feasible because of the high contamination risks and low isolation success rates, UCB seems a promising alternative MSC source, especially when considering allogeneic MSC use.

## Introduction

In the past decade, mesenchymal stromal cells (MSCs) from humans as well as a variety of other animal species have received tremendous attention because of their potential for cell-based therapies based on their immunomodulatory and anti-inflammatory capacities. The low immunogenicity of MSCs, indicated by the absence of the expression of major histocompatibility complex (MHC) class II and co-stimulatory molecules CD40, CD80, and CD86, enables their use in clinical allogeneic applications [1]. Moreover, numerous soluble factors play an essential role in the regulation of MSC-mediated immunosuppression by inhibiting T-cell proliferation [2,3]. It has been suggested that the release of these secreted factors like hepatocyte growth factor (HGF) and transforming growth factor-beta (TGF-β) and the induction of indoleamine 2,3-dioxygenase (IDO) mediate the immunosuppressive effects of MSCs [4,5]. Moreover, tumor necrosis factor-alpha (TNFα) and interferon-gamma (IFNγ) stimulate the secretion of immunomodulatory mediators from MSCs [3,6].

In general, most cell-based therapies consist of the use of autologous MSCs. Nevertheless, given their immunosuppressive capacities, MSCs seem perfectly suited for cellular therapy in allogeneic settings [4]. An allogeneic source would provide an off-the-shelf, more standardized, and readily available product without the inherent lag period associated with isolation and expansion of autologous MSCs [7,8]. Some preliminary studies have suggested that equine allogeneic MSCs can be used without eliciting an apparent cell-mediated immune response in horses. Indeed, a study by Guest and colleagues [9] found that the injection of allogeneic bone marrow (BM)-derived MSCs did not cause any observable cell-mediated immune response from the host. In a later study by Carrade and colleagues [10], no significant differences could be demonstrated either in the type or in the degree of inflammation when using autologous or related or unrelated allogeneic cells. These findings indicate that allogeneic MSCs may be both a safe and practical alternative treatment option for equine orthopedic injuries.

Traditionally, BM and adipose tissue have been used to harvest equine MSCs. Issues concerning the ease of isolation, expansion characteristics, and donor site complications warrant the search for alternative sources [11]. The MSCs from umbilical cord blood (UCB) and umbilical cord matrix (UCM) can be easily collected at parturition without harming the mare or the foal, expanded and subsequently cryopreserved, and as such be readily available at the time of injury [8]. Thus, by using these non-invasive sources, the optimal time for treatment can be determined by the clinician in sharp contrast to the use of MSCs from BM or adipose tissue, for which the time for cellular expansion must be taken into account [12]. Nevertheless, cryopreservation of MSCs implies a long-term, expensive storage [12]. Peripheral blood (PB) is also considered an attractive alternative since the collection of a venous blood sample is a minimally invasive and easy procedure that can be performed at any time [13,14].

Therefore, the aim of the present study was to analyze and compare these three attractive sources of equine MSCs (that is, UCB, UCM, and PB). To this end, MSCs were characterized both functionally as well as immunophenotypically by analyzing the following parameters: (i) success rate of isolation, (ii) proliferation capacity, (iii) tri-lineage differentiation ability, (iv) immunophenotypical protein profile, and (v) immunomodulatory mRNA profile.

## Materials and methods

### Media

The culture medium for UCB and PB cultures contained low-glucose Dulbecco’s modified Eagle’s medium (DMEM) (Invitrogen Corporation, Carlsbad, CA, USA), 30% fetal calf serum (FCS) (Gibco-BRL, now part of Invitrogen, Ghent, Belgium), 10^−7^ M low dexamethazone, 50 μg/mL gentamycine, 10 μL/mL antibiotic antimycotic solution (consisting of 10,000 units penicillin, 10 mg streptomycin, and 25 μg amphotericin B per mL), 250 ng/mL fungizone (all from Sigma-Aldrich, Bornem, Belgium) and 2 mM ultraglutamine (Invitrogen, Ghent, Belgium), based on the medium described by Koch and colleagues [15]. The expansion medium was identical to the culture medium but without dexamethasone. The UCM culture medium contained low-glucose DMEM, 15% FCS, 50 μg/mL gentamycine, 10 μL/mL antibiotic antimycotic solution, and 250 ng/mL fungizone. Osteogenic medium consisted of low-glucose DMEM, 10% FCS, 0.2 mM L-ascorbic acid-2-phosphate (Fluka: part of Sigma-Aldrich), 100 nM dexamethasone, 10 mM β-glycerophosphate, 50 μg/mL gentamycine, and 10 μL/mL antibiotic antimycotic solution. Chondrogenic medium was based on the basal differentiation medium from Lonza, Basel, Switzerland, complemented with 10 ng/mL TGF-β3 (Sigma-Aldrich). Adipogenic induction medium contained low-glucose DMEM, 1 μM dexamethasone, 0.5 mM 3-isobutyl-1-methylxanthine, 10 μg/mL rh-insuline, 0.2 mM indomethacin, 15% rabbit serum (all from Sigma-Aldrich), 50 μg/mL gentamycine, and 10 μl/mL antibiotic antimycotic solution. The adipogenic maintenance medium was identical to the adipogenic induction medium but without dexamethasone, indomethacin, and 3-isobutyl-1-methylxanthine.

### Collection of umbilical cord blood, umbilical cord matrix, and peripheral blood

UCB was collected from warmblood full-term born foals immediately after birth. After clamping and disinfecting of the umbilical cord with 70% alcohol, the umbilical vein was punctured immediately after birth and UCB was drained by gravity into a standard blood donor bag (Terumo, Heverlee, Belgium®; Terumo BCT, Lakewood, CO, USA) and subsequently stored at 4°C. Samples were processed only if (i) at least 150 mL UCB was collected, (ii) storage time was less than 15 hours, and (iii) no signs of coagulation or hemolysis were present [16]. Once the umbilical cord was ruptured spontaneously, a clamp was placed on each end of the amniotic part, after which the umbilical cord was rinsed with tap water and iodine soap to remove the gross contamination [17] and disinfected with 70% alcohol. Subsequently, a 5-cm-long piece was obtained from the middle of the disinfected umbilical cord with a sterile scalpel blade and stored in phosphate-buffered saline containing 50 μg/mL gentamicin at 4°C. At the same moment, PB from the vena jugularis of the mares (ages between 4 and 16 years) was collected into two vacuum blood tubes containing heparin as anti-coagulant and stored at 4°C until further processing. The study was approved by the Ethical Committee of the Faculty of Veterinary Medicine of Ghent University (EC2010/147).

### Isolation and culture of equine mesenchymal stromal cells

Equine MSCs derived from UCB and PB were isolated and cultured as previously described [18]. Briefly, UCB or PB was centrifuged at 1,000 *g* for 20 minutes at room temperature (RT). After diluting of the obtained buffy coat fraction 1:1 (vol:vol) with Hanks’ balanced salt solution (HBSS), the cell suspension was gently layered on an equal volume of Percoll® (density 1.080 g/mL; GE Healthcare, Little Chalfont, Buckinghamshire, UK) and centrifuged for 15 minutes at 600 *g* at RT. The interphase, containing the mononuclear cell (MNC) fraction, was collected and washed three times with HBSS by centrifuging 10 minutes at 200 *g* at RT. Cell viability and concentration were determined by trypan blue exclusion by using the improved Neubauer hemocytometer. The MNCs were seeded in culture medium at 1 × 10^6^ cells/mL in uncoated T-25 culture flasks using 12-mL culture medium and incubated at 37.5°C in a humidified atmosphere containing 5% CO_2_. Non-adherent cells were removed the following day by completely replacing the culture medium, after which the medium was exchanged twice a week. When numerous colonies of adherent cells were observed, cells were detached by using 0.083% trypsin-ethylenediaminetetraacetic acid (EDTA) (Sigma-Aldrich, Bornem, Belgium) and further passaged in expansion medium, irrespectively of their source.

Equine MSCs derived from UCM were also isolated and cultured as previously described [19]. Briefly, the umbilical cord was disinfected with Octeniderm® antiseptic spray (Schülke & Mayr, Norderstedt, Germany) in a laminar flow hood, after which the umbilical arteries and vein were removed. The UCM was minced finely (0.5 cm^2^) by using sterile scissors in a sterile glass Petri dish containing UCM culture medium. Subsequently, the explants were transferred to a T-25 culture flask in 6 mL UCM culture medium and incubated at 37.5°C in a humidified atmosphere containing 5% CO_2_. The explants were left undisturbed for 3 days, after which the medium was exchanged. Ten days after the start of the culture, the explants were removed and culture medium was exchanged again. Cells were detached with 0.083% trypsin-EDTA when numerous colonies of adherent cells were observed and further passaged in expansion medium.

### Proliferation studies

MSCs isolated from UCB, UCM, or PB were followed during five subsequent passages and for each passage, cell concentration was determined in order to calculate the cell doubling number (CDN) and the population doubling time (PDT) by using following formula: CDN = ln (Nf/Ni)/ln 2, where Nf is the final number of cells and Ni the initial number of cells, and PDT = cell culture time (in days)/CDN.

### Tri-lineage differentiation

After two passages, approximately 1 × 10^6^ undifferentiated MSCs were used to perform the tri-lineage differentiation experiments, exactly as previously described [14,18]. Non-induced cells in expansion medium were used as negative controls. Osteogenic differentiation was performed in six-well culture dishes with approximately 3,000 undifferentiated MSCs/cm^2^ which were cultured in expansion medium until 90% to 100% confluency was reached. Subsequently, osteogenic differentiation was induced with osteogenic medium that was exchanged twice a week, and evaluated after 20 days of culture by using the Alizarin Red S histological staining as well as by detecting alkaline phosphatase activity (Millipore, Overijse, Belgium). Chondrogenic differentiation was performed by using a micromass culture system; that is, 2.5 × 10^6^ cells were centrifuged in 15-mL Falcon tubes at 150 *g* for 5 minutes at RT, after which the chondrogenic medium was added without disturbing the cell pellet. The medium was exchanged every 3 or 4 days during 3 weeks, after which the chondrogenic differentiation was evaluated by the Alcian blue histological staining. To initiate the adipogenic differentiation, 2.1 × 10^4^ undifferentiated MSC/cm^2^ were seeded in six-well culture dishes and cultured until 100% confluency. Subsequently, cells were exposed to four cycles of 72-hour culturing in the adipogenic induction medium and 24 hours of culturing in the adipogenic maintenance medium, followed by five consecutive days of culturing in adipogenic maintenance medium. Oil Red O histological staining was used to detect the intracellular accumulation of lipid droplets.

### Immunophenotypical protein profile as determined by multi-color flow cytometry

Following the criteria of the International Society for Cellular Therapy, human MSCs need to express CD29, CD44, CD73, CD90, and CD105 and lack expression of CD14, CD34, CD45, CD79α, and MHC-II [20]. Undifferentiated equine MSCs from the third/fourth passage were immunophenotyped by using multicolor flow cytometry, exactly as previously described [21]. A detailed description of monoclonal antibody (mAb) clones and dilutions is given in Table 1. Commercially available mAbs were validated for recognizing equine epitopes by using freshly isolated equine MNCs, lymphocytes, or primary endothelial cells as appropriate positive control cells (Table 1) [21]. The following combinations of marker panels were assessed: CD29-Alexa488/MHC II-RPE/CD44-APC/7-AAD (subset 1), CD105-RPE/CD90-Alexa647/7-AAD (subset 2), CD45-Alexa488/CD73-RPE/7-AAD (subset 3), and the monocyte marker-Alexa488/CD79α-Alexa647 (subset 4). To identify the viable cells, 7-AAD was used in the first three subsets. The presence of MHC-I on the cell surface of the undifferentiated MSCs was analyzed separately as this mAb could not be included in any of the subsets.

**Table 1:** Overview of the marker panels of primary monoclonal antibodies and 7-AAD to immunophenotype equine MSC

	**Subset**	**Marker**	**Company**	**Clone**	**Sec Ab**	**Dilution**	**Positive control**
Multicolor FCM	1	CD29-Alexa488^+^	Biolegend	TS2/16		1:20	MNCs
		MHC-II^−^	Serotec	CVS20	RPE	1:50	MNCs
		7-AAD^−^	Calbiochem				
		CD44-APC^+^	BD	IM7		1:20	MNCs
	2	CD105-RPE^+^	Serotec	SN6		1:10	Endothelial cells
		7-AAD^−^	Calbiochem				
		CD90^+^	VMRD	DH24A	Alexa647	1:100	MNCs
	3	CD45-Alexa488^−^	Serotec	F10-89-4		1:5	MNCs
		CD73^+^	Abcam	10f1	RPE	1:5	Lymphocytes
		7-AAD^−^	Calbiochem				
	4	Monocyte-Alexa488^−^	Serotec	MAC387		1:2.5	MNCs
		CD79α-Alexa647^−^	Serotec	HM57		1:2.5	MNCs
Single-color FCM		MHC-I^+^	VMRD	PT85A	RPE	1:66	MNCs
Secondary Ab	1 and 3	Sheep anti-mouse IgG-RPE	Sigma-Aldrich			1:20	
	2	Goat anti-mouse IgG-Alexa647	Invitrogen Corporation			1:200	
Isotype controls	1 and 4	Mouse IgG1-Alexa488	Biolegend			1:20	
	1-3	Mouse IgG1-RPE	Biolegend			1:10	
	1	Rat IgG2b-APC	Biolegend			1:20	
	2	Mouse IgM	BD		Alexa647	1:50	
	3	Mouse IgG2-Alexa488	Biolegend			1:20	
	4	Mouse IgG1-Alexa647	Biolegend			1:100	

7-AAD, 7-aminoactinomycin D; FCM, fluorescence correlation microscopy; MHC, major histocompatibility complex; MNC, mononuclear cell; Sec Ab, secondary antibody.

### Immunomodulatory mRNA profile as determined by quantitative polymerase chain reaction

For each source, 1 × 10^6^ MSCs from the fourth passage were stored in freezing medium, consisting of high-glucose DMEM, 10% FCS, and 20% dimethylsulphoxide (DMSO) (Sigma-Aldrich), at −80°C until RNA extraction. Total RNA was isolated with the RNeasy Mini Kit (Qiagen, Hilden, Germany) in accordance with the instructions of the manufacturer, including an on-column DNAse treatment for 30 minutes with RNase free DNAse (Qiagen). After a minus RT control to check for contaminating genomic DNA, 100 ng of RNA was reverse-transcribed into cDNA by using the Iscript advanced cDNA synthesis kit (Bio-Rad, Nazareth-Eke, Belgium).

**Figure 1:**
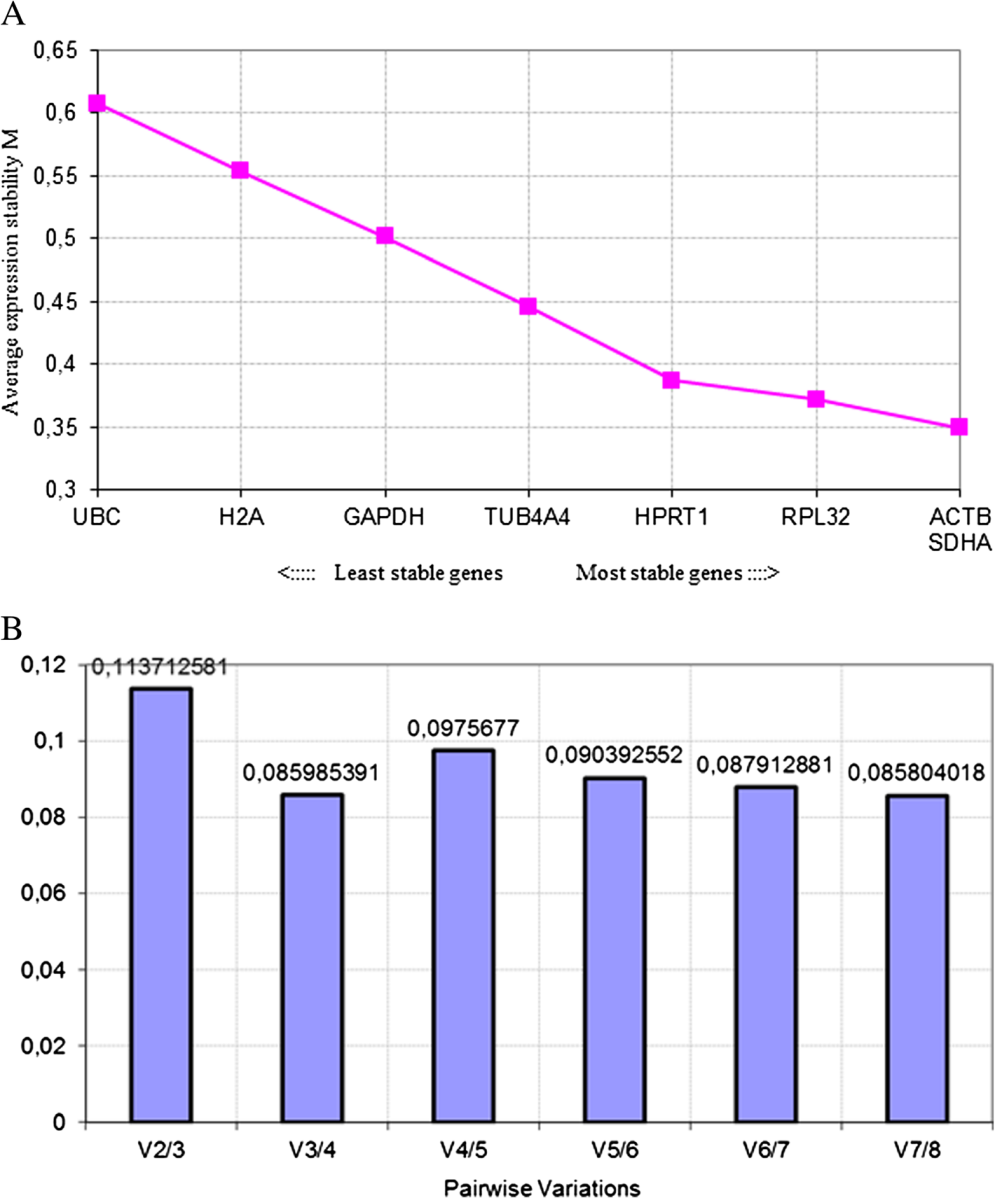
**Determination of the stability and number of reference genes. (A)** Average expression stability values of eight commonly used reference genes. **(B)** Determination of the optimal number of reference genes to be used for normalization. The Y-axis represents the pairwise variation value V between the normalization factors NFn and NFn + 1. Pairwise variation values are calculated for every series of NFn and NFn + 1 normalization factors, reflecting the effect of adding an (n + 1)th gene.

In a preliminary experiment, the expression stability of eight commonly used reference genes [22,23] was determined in the MSC samples by using geNorm software [24] (Table 2). Applying this software program indicated beta actin (*ACTB*), succinate dehydrogenase complex, subunit A (*SDHA*), ribosomal protein L32 (*RPL32*), and hypoxanthine phosphoribosyl-transferase 1 (*HPRT1*) as the most stable genes in MSC samples, since the average expression stability values (or M values) of these genes ranged between 0.35 and 0.4, indicating a very good stability (Figure 1A). The pairwise variation calculation showed that at least four reference genes should be used for normalization (Figure 1B).

**Table 2:** Primer sequences used for the real-time polymerase chain reaction studies

	**Gene**	**Name**	**Primer sequence 5′ → 3′**	**Amplicon size, bp**	**Ta, °C**	**Efficiency, %**	**Correlation**
Reference genes	*ACTB*	Beta actin	CCAGCACGATGAAGATCAAG	88	60	98	0.998
			GTGGACAATGAGGCCAGAAT				
	*GAPDH*	Glyceraldehyde-3-phosphate dehydrogenase	CAGAACATCATCCCTGCTTC	187	59	100	0.998
			ATGCCTGCTTCACCACCTTC				
	*H2A*	Histone H2A type 1-C	ATATTCAGGCCGTGCTGCT	105	60	100	0.999
			TTTGGGTTTCAAAGCGTTTC				
	*HPRT1*	Hypoxanthine phosphoribosyl-transferase 1	GGCAAAACAATGCAAACCTT	163	57	100	0.994
			CAAGGGCATATCCTACGACAA				
	*SDHA*	Succinate dehydrogenase complex, subunit A	TCCATCGCATAAGAGCAAAG	159	59	99	0.997
			GGTGGAACTGAACGAACTCC				
	*RPL32*	Ribosomal protein L32	AGCCATCTACTCGGCGTCA	149	60	100	0.995
			TCCAATGCCTCTGGGTTTC				
	*UBC*	Ubiquitin C	GCAAGACCATCACCCTGGA	206	60	97	0.996
			CTAACAGCCACCCCTGAGAC				
	*TUBA4A*	Tubulin, alpha 4a	GCCCTACAACTCCATCCTGA	78	60	100	0.999
			ATGGCTTCATTGTCCACCA				
Test genes	*CD40*	Cluster of differentiation 40	CAGGAAAGAAACTGGTGAATG	180	62	106	0.993
			AAGTGGCGTCTGTTGTTGAG				
	*CD80*	Cluster of differentiation 80	CACCTTCACCGACATCACC	106	62-64	102	0.995
			AGACCCCCTTTCGCTCTTC				
	*CD86*	Cluster of differentiation 86	AGTATAAAGGCCGCACAAGC CCTTGGGTAGATGAGCAGGT	247	63	95	0.990
	*HGF*	Hepatocyte growth factor	TGCATTCAAGGTCAAGGAGA TTTTGGAATTTGGGAGCAGT	234	63	84	0.983
	*IDO*	Indoleamine 2,3-dioxygenase	ACAACATCAGGACCAGGACAC TCCAGACGCCTTCATAGAG	198	61-64	72	0.957
	*TGFβ*	Transforming growth factor-β	GGAATGGCTGTCCTTTGATG CGGAGTGTGTTATCTTTGCTGTC	120	61-64	92	0.997
	*TNFα*	Tumor necrosis factor-α	GCCTCAGCCTCTTCTCCTTC GGCTTGTCACTTGGGGTTC	172	62-64	113	0.999

Ta, annealing temperature.

Primers for the immunomodulatory (co)factors CD40, CD80, CD86, HGF, IDO, TGFβ, and TNFα were designed by means of Primer3 software [25], based on horse sequences found in the National Center for Biotechnology Information (NCBI) GenBank [26] (Table 2). Primers were, where possible, selected over intron-exon boundaries, tested by using a Basic Local Alignment Search Tool (BLAST) analysis against the NCBI database and tested for secondary structure formation by using MFold [27]. The optimal quantitative PCR (qPCR) conditions were determined on cDNA of mixed equine tissues consisting of PB, MSCs, and endothelial cells.

qPCR analyses were performed in accordance with the MIQE (Minimum Information for Publication of Quantitative Real-Time PCR Experiments) guidelines [28] on the CFX96 Touch Real-Time PCR Detection System (Bio-Rad) by using the SsoAdvanced SYBR green supermix (Bio-Rad) and 0.2 mL thin-walled 96-well PCR plates (Bio-Rad). Each 10 μL qPCR reaction consisted of 5 μL of SsoAdvanced SYBR green master mix, 200 nM of each specific primer, and 2 μL of 10× diluted cDNA. The PCR program started with an initial 3-minute denaturation at 95°C to activate the DNA polymerase, followed by 45 cycles of denaturation at 95°C for 20 seconds and a combined primer annealing/extension at the specific annealing temperature for 40 seconds during which fluorescence was measured. The level of mRNA expression was assessed on the basis of the Cq value. A melt curve analysis followed by agarose gel electrophoresis was performed to test for primer-dimer formation and specificity of the amplicons. Each qPCR was executed in duplicate, no template and no RT controls were included (Cq > 45), and a 6-point, fivefold serial dilution series made of cDNA isolated from equine blood, MSCs, and endothelial cells gave information about the PCR efficiencies, the correlation coefficients (Table 2), the slopes, and the Y-intercepts of the assays. Cq values were converted into raw data and analyzed by the ΔΔCq method as described by Hellemans and colleagues [29] and normalized by using the geometric mean of the four most stably expressed reference genes.

### Statistical analysis

Data were presented as mean ± standard deviation. Linear regression models were fit to determine the association between the source of MSCs (UCB, UCM, PB) and (i) the moment of first observation, (ii) the moment of first passage, (iii) cell proliferation data (that is, CDN and PDT), (iv) the expression of markers related to cell immunogenicity (that is, MHC-I and MHC-II), and (v) the mRNA profile of all immunomodulatory factors, except for HGF as no normal distribution could be obtained for the latter variable. To evaluate the association between the source of MSCs and the mRNA expression of HGF, the non-parametric Kruskal-Wallis test was performed instead. To minimalize inter-horse variability, samples of PB, UCB, and UCM were at least matched within the same mare and for UCB and UCM even within the same foal from that specific mare. In all linear regression models, mare was forced into the model to exclude mare-specific effects. Normality was checked by the Kolmogorov-Smirnov test. To approximate normality, a reciprocal (CD105) or arcsin-transformation (CD44, CD90, subset 2, CD79, MHC-1, monocyte marker, subset 4) of the different markers was performed. A reciprocal transformation was also used to obtain a normal distribution of the PDT. In all models, statistical significance was assessed at *P*<0.05. The fit of the linear regression models was evaluated by examination of the normal probability plots of residuals and by inspection of the residuals plotted against the predicted values. Least square means were calculated. All analyses were performed by using SPSS 19.0 (SPSS, Inc., Chicago, IL, USA).

## Results

### Success rate of isolating equine mesenchymal stromal cells

UCB, UCM, and PB samples were collected from six mares with a normal parturition, and putative equine MSCs could be isolated from all three sources (Table 3). All adherent cell populations had the typical spindle-shaped fibroblast-like cells morphology, irrespectively of the source used. An isolation success rate of 100% was found for MSC isolation from both UCB and PB. For the isolation of MSCs from UCM, however, MSCs could be obtained from two out of the six samples because of bacterial contamination issues (Table 3).

**Table 3:** Descriptive statistics of the success rate ± standard deviation (SD, %) and linear regression model describing the effect of MSC source on the moment of first observation ± SD (days) and moment of first passage ± SD (days) of the isolation of putative equine MSCs from 6 UCB, PB, and UCM respectively

**Source**	**Success rate, percentage**	**First observation, days**	**First passage, days**
UCB	100	8.5 ± 1.9^a^	15.8 ± 2.4^a^
PB	100	14.5 ± 1.9	19.7 ± 2.4
UCM	33.3	14.5 ± 2.2	21.5 ± 4.8

^a^Umbilical cord blood (UCB) significantly different from peripheral blood (PB) and umbilical cord matrix (UCM) at *P*<0.01.

Interestingly, adherent cells could be observed in the UCB samples on average as early as 8 days after culturing, in contrast to UCM and PB samples, in which adherent cells were first spotted around 14 days (*P*<0.01) (Table 3).

### Cell proliferation

**Figure 2:**
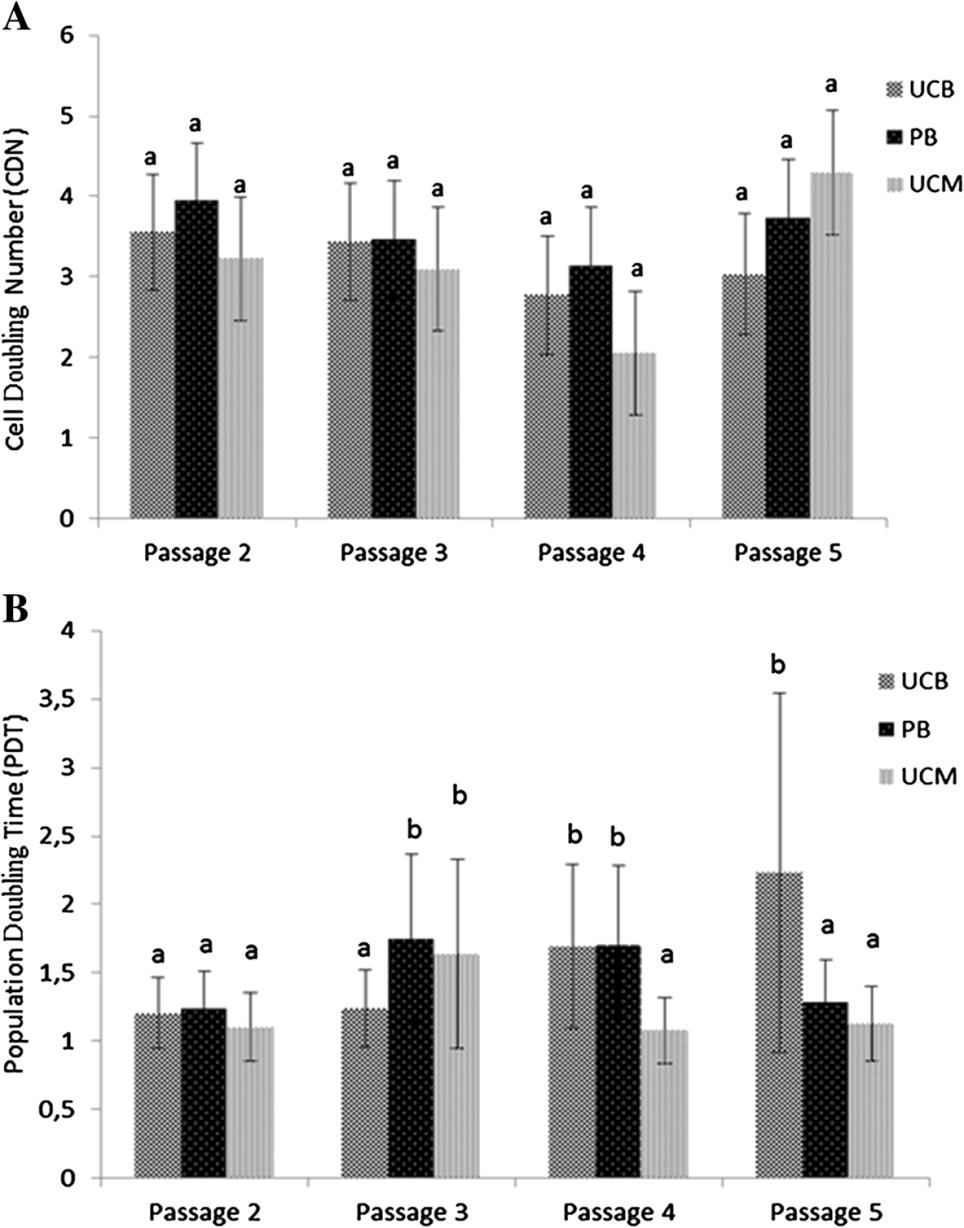
**Proliferation data of MSCs derived from UCB, PB, and UCM for passages 2 to 5, expressed by the least square means ± SD of the CDN (A) and the back-transformed least square means of the PDT (B).** Different superscripts (a,b) denote statistically significant differences between either sources or passages not sharing the same superscript.

Kinetic parameters such as the CDN and PDT enable a good monitoring of a culture during serial passage by calculating cell yields (CDN) and growth rates (PDT). To assess cell proliferation, CDN and PDT were determined for MSCs from all three sources between passages 2 and 5, and the overall results indicated that MSCs were able to rapidly divide *in vitro*, irrespectively of their source. With regard to the CDN data, no statistically significant differences were observed between sources (Figure 2A). In addition, PDT values did not substantially differ between the different sources with the exception of passage 3, in which the PDT of UCB-derived MSCs (1.24 ± 0.28 days) was significantly lower than the PDT of PB- and UCM-derived MSCs (1.75 ± 0.61 days and 1.64 ± 0.69 days, respectively) (Figure 2B) (*P*<0.05). This was shown to be primarily due to the source-specific cell division patterns (Table 4). The PDT of UCB increased from 1.20 ± 0.26 at passage 2 to 2.23 ± 1.31 at passage 5, while the PDT of PB and UCM, respectively, steeply increased from 1.24 ± 0.27 and 1.10 ± 0.25 at passage 2 to 1.75 ± 0.61 and 1.64 ± 0.69 at passage 3 and then subsequently decreased to 1.71 ± 0.58 and 1.08 ± 0.24 at passage 4 and to 1.29 ± 0.31 and 1.13 ± 0.27 at passage 5, respectively (Figure 2B). In conclusion, the PDT for UCB-derived MSCs gradually increased while the PDT for PB- and UCM-derived MSCs was initially higher in comparison with UCB-derived MSCs but then substantially decreased.

**Table 4:** Linear regression model describing the effect of the source of equine MSC and the passage on the reciprocal transformed PDT

**Independent variable**	**n**^**a**^	**Estimate**^**b**^	**SE**^**c**^	**LSM**^**d**^	***P *** **value**
Mare	6	…	…	…	<0.001
Source					0.12
Umbilical cord blood	6	ref.	…	1.49	…
Peripheral blood	6	0.02	0.05	1.47	0.75
Umbilical cord matrix	2	0.16	0.08	1.21	0.04
Passage					0.03
2	14	ref.	…	1.18	…
3	12	−0.18	0.07	1.51	0.01
4	11	−0.15	0.07	1.42	0.03
5	10	−0.14	0.07	1.42	0.04
Source x passage^e^	…	…	…	…	<0.001

^a^Number of observations. ^b^Reciprocal transformation of population doubling time. ^c^Standard error of the mean. ^d^Back-transformed least square means. ^e^Estimates are not shown. See Figure 2 for least square mean values for different comparisons.

### Tri-lineage differentiation potential

**Figure 3:**
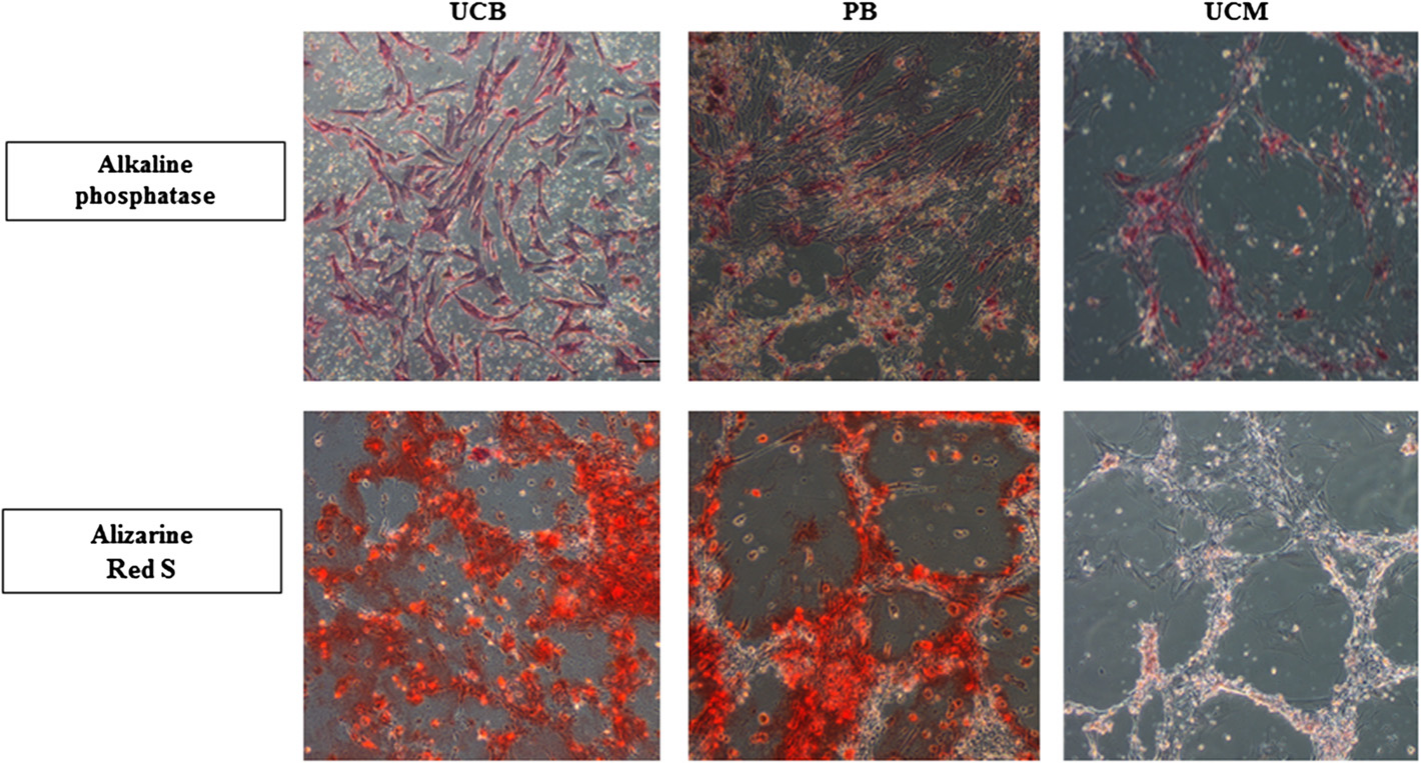
**Osteogenic differentiation potential.** Representative example of isolated equine umbilical cord blood- (UCB-), peripheral blood- (PB-), and umbilical cord matrix- (UCM-) derived mesenchymal stromal cells (MSCs) which were able to differentiate toward the osteogenic lineage, as demonstrated by an increased alkaline phosphatase activity and the Alizarin Red S staining. MSCs derived from UCM are staining positive for alkaline phosphatase activity but negative for Alizarin Red S. Magnification 10×.

**Figure 4:**
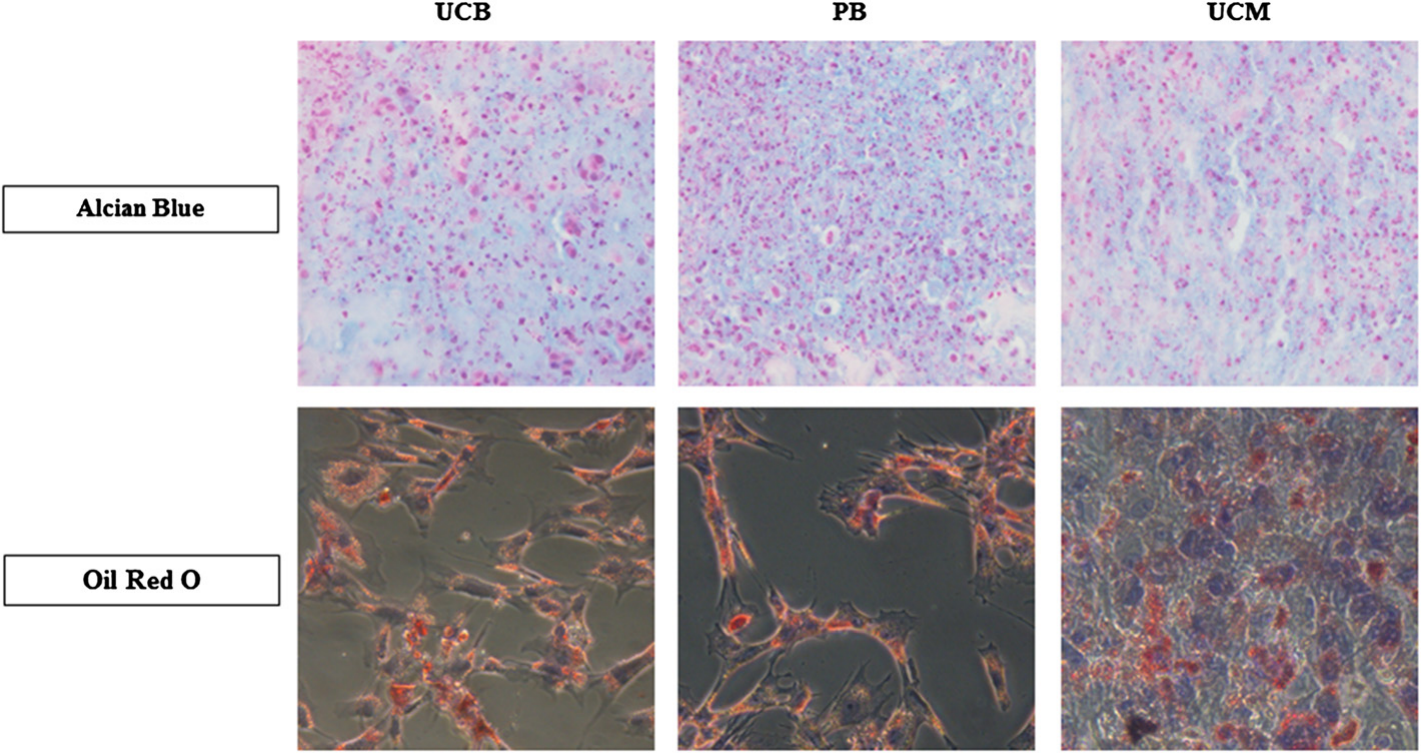
**Chondrogenic and adipogenic differentiation potential.** A representative example of isolated equine mesenchymal stromal cells (MSCs) derived from umbilical cord blood (UCB), peripheral blood (PB), and umbilical cord matrix (UCM) was shown to demonstrate the chondrogenic and adipogenic differentiation. Magnification 40× (chondrogenic differentiation) and 20× (adipogenic differentiation).

Osteogenic differentiation was confirmed in all six UCB and PB samples by an increased expression of alkaline phosphatase activity as well as an Alizarin Red S-positive histological staining (Figure 3), with the exception of the set of UCB/PB samples from one mare, in which no calcium deposits could be detected with Alizarin red S (data not shown). In MSCs isolated from the two non-contaminated UCM samples, an increased alkaline phosphatase activity could be clearly demonstrated but these samples were negative for the Alizarin Red S staining (Figure 3). No difference in chondrogenic and adipogenic differentiation potential was noticed for MSCs derived from either source, and all MSCs readily differentiated toward chondrocytes, as evaluated by a positive Alcian blue staining, and toward adipocytes, as evaluated by a positive Oil Red O staining (Figure 4).

### Immunophenotypical protein profile

An overview of the results obtained for the expression of the different markers on equine MSCs derived from either source is given in Table 5. Briefly, although significant variations were observed in the percentage of some markers depending on the source, the overall tendency was similar with a high expression of markers known to be expressed on MSCs and a negligible expression of markers known to be absent in MSCs (Table 5). However, since both the absolute difference between the three sources and the variation within each source were very small, the biological relevance of these statistically significant differences could be questioned. Indeed, such differences in marker expression might just represent the biological diversity among the animals, as suggested previously by Pascucci and colleagues [30]. In addition, the variable expression of CD105 observed in the present study is consistent with our previous studies and has been extensively discussed [18,21,31].

**Table 5:** Immunophenotypical characterization of equine mesenchymal stromal cells derived from umbilical cord blood, peripheral blood, and umbilical cord matrix, expressed as the percentage of cells positive for each marker

**Marker**	**UCB**	**PB**	**UCM**
CD29^pos^	99.6 ± 0.2^a^	98.2 ± 0.9^a^	98.4 ± 1.0^a^
CD44^pos^	99.2 ± 0.2^b^	96.6 ± 4.1	97.3 ± 1.1
CD73^pos^	0.5 ± 0.5^c,d^	0.8 ± 0.5	0.5 ± 0.1
CD90^pos^	99.4 ± 0.3	97.8 ± 2.4	66.9 ± 27.6^a^
CD105^pos^	2.7 ± 3.4	1.2 ± 1.6	46.2 ± 64.3
MHC-I^pos^	80.3 ± 12.9	62.7 ± 24.8^c^	77.6 ± 11.8
CD45^neg^	0.5 ± 0.3	1.8 ± 0.9	1.1 ± 1.4
CD79α^neg^	0.1 ± 0.1^c^	1.0 ± 1.0^c^	0.1 ± 0^*^
Monocyte marker^neg^	0.2 ± 0.1^b,d^	1.3 ± 1.2	1.0 ± 1.3
MHC-II^neg^	0.5 ± 0.5	0.8 ± 0.7	0.8 ± 0.6

Indicated source is significantly different from both other sources at least at ^a^*P*<0.001, ^b^*P*<0.01, ^c^*P*<0.05. ^d^Only significantly different from peripheral blood (PB). Data are presented as average ± standard deviation (n = 6, except for umbilical cord matrix (UCM), n = 2). UCB, umbilical cord blood.

When evaluating vitality, using 7-AAD as a cell viability stain, a consistently higher amount of dead cells was observed in PB-derived MSC cultures (13.3% ± 1.3%) compared with UCB-derived (7.7% ± 1.3%) or UCM-derived (5.4% ± 0.8%) MSCs (*P*<0.001).

### Immunomodulatory mRNA profile

**Figure 5:**
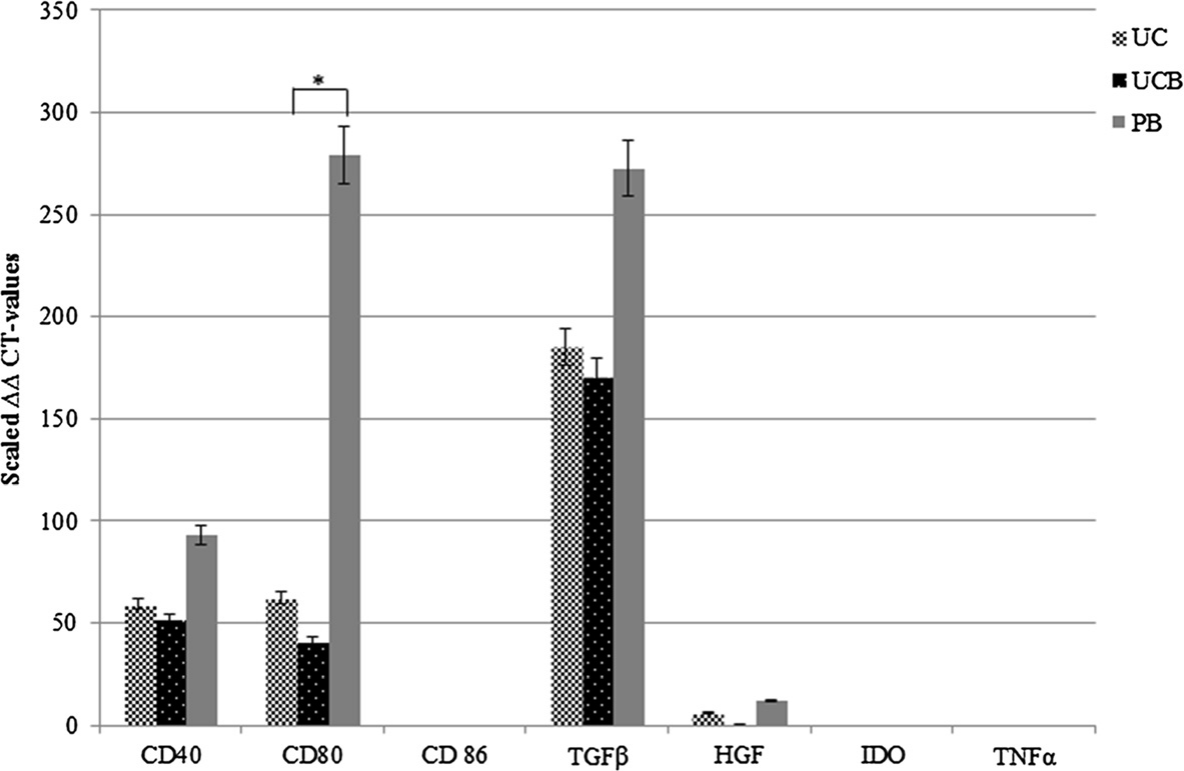
**Differential gene expression in UCB-, UCM-, PB-derived MSCs, as determined by RT-qPCR.** Mean normalized expression and error bars with 5% value are displayed. *P<0.05.

Finally, we also wanted to study and compare the expression of several immunomodulatory factors, which are well studied in human MSC immunobiology [4], in our equine MSCs obtained from the three sources. To this end, mRNA expression of the different factors was evaluated by reverse transcription-quantitative PCR (RT-qPCR). Equine MSCs showed a moderate to strong expression of the co-stimulatory molecules CD40 and CD80, irrespectively of the source, with a significantly lower expression for CD80 on UCB-derived MSCs compared with PB-derived MSCs (*P*<0.05) (Figure 5). In contrast, no CD86 expression was found in MSCs from all three sources (Figure 5).

Additionally, the equine MSCs were examined for the expression of genes encoding several cytokines and growth factors such as HGF, IDO, TGFβ, and TNFα. A strong expression was observed for TGFβ, followed by a moderate to weak expression of HGF, again irrespectively of source (Figure 5). In contrast, no expression was noted for IDO and TNFα in all samples tested (Figure 5).

## Discussion

In this study, we aimed to thoroughly evaluate and compare three minimally invasive sources to obtain equine MSCs (that is, UCB, UCM, and PB) in order to define the most valuable source for regenerative purposes. This included assessment of success rate of isolation, proliferation potential, and differentiation capacities and evaluation of their immunophenotypical and immunomodulatory profiles. The latter profile may be important in view of envisaged allogeneic future applications. To minimalize animal-dependent influences between the different sources, the samples of the three tissue sources were at least matched within the same mare and for UCB and UCM even within the same foal from that specific mare. As such, we significantly reduced the large variability in age and breed of the horses [32]. As an overall conclusion, we propose UCB as the most valuable, non-invasive source for equine MSCs on the basis of the following observations. Firstly, an isolation success rate of 100% was found for MSC isolation from both UCB and PB, which might indicate that the isolation methods and culture conditions for equine MSCs from these sources have been optimized. Since obtaining contamination-free UCM cultures was found to be problematic and fungal or bacterial contamination has also been described in other studies using UCM as a source for MSCs [33-35], we concluded that this tissue is a less appealing source for equine MSCs. Secondly, MSCs were first observed in the UCB samples and this could not be explained by the number of peripheral blood mononuclear cells (PBMCs) originally seeded, since this was kept constant for all blood samples tested. It might, however, be explained by the fact that the concentration of circulating MSCs in PB is likely very low [13,32,36,37]. As the explants of the UCM were immediately transferred to culture flasks and MSCs were supposed to migrate from these explants, it is not known how many UCM-derived cells were initially seeded. However, one might suppose that the cells from the UCM need extra time to migrate out of the explants instead of just adhering to the culture flask, which might explain why MSCs were observed in a later stage when compared with UCB. Thirdly, regarding the osteogenic differentiation, UCM-derived MSCs did not produce a mineralized matrix, being a critical feature of the more mature stage of osteogenesis, and thus did not differentiate toward fully matured osteocytes. Increasing the serum concentration in the osteogenic differentiation medium from 10% to 20% might have enhanced the osteogenic potential of our UCM-derived MSCs, as previously described by Toupadakis and colleagues [11], but this was not evaluated in our present study, because we wanted to keep the osteogenic medium identical for all three MSC sources tested.

Allogeneic applications for regenerative therapies are an attractive approach since allogeneic MSCs would provide an off-the-shelf, more standardized, and readily available product without the inherent lag period associated with isolation and expansion of autologous MSCs [7]. A potential drawback of using allogeneic MSCs, however, might be their immunogeneic properties which could lead to an immune response in the acceptor host and, hence, rejection reactions. For equine MSCs, however, not much information regarding their immunogenic/immunomodulatory profiles is available. Therefore, we initiated an evaluation and comparison of important immunological factors in equine MSCs from the three sources tested in this study.

First, the expressions at the protein level of the histocompatibility markers MHC-I and MHC-II were evaluated by flow cytometry. Equine MSCs from all sources showed a moderate to high expression of MHC-I, ranging from 62.7% ± 24.8% for PB to 80.3% ± 12.9% for UCB, while the expression of MHC-II was lacking in all MSC samples, which is similar to what has been previously described [10,38]. In a study by Guest and colleagues [38], a lower expression of MHC-I on UCB-derived MSCs was found when compared with BM-derived MSCs. However, the presence of MHC-I on MSCs seems less important when evaluating the ability of MSCs to elicit acute graft rejection or cell-mediated delayed-type hypersensitivity responses in allogeneic settings, since it was recently demonstrated by Carrade and colleagues [10] that the intradermal injection of allogeneic MSCs generated only a limited, self-resolving dermal histopathologic response, similar to the injection of saline. The same study by Guest and colleagues [38] found no expression of MHC-II on both BM-derived and UCB-derived MSCs. After treatment with IFN-γ, MHC-II expression was induced on BM-derived MSCs but not on UCB-derived MSCs, suggesting that MSCs from neonatal sources are less immunogenically established than MSCs from adult sources. Granted, IFN-γ stimulation of equine MSCs was not performed in our present study, but future experiments are planned to evaluate whether the absence of MHC-II on these cells will translate into their inability to stimulate host cells *in vitro* as well as *in vivo*. Hereby, we anticipate that our equine MSCs will not be immunogenic or will be only low immunogenic, based on *in vitro* studies which have demonstrated that human BM-derived MSCs induced no proliferative response in mixed lymphocyte reactions, even after IFN-γ stimulation and induction of MHC-II expression [39].

Additionally, the expression of the co-stimulatory molecules CD40, CD80, and CD86 was evaluated by using qPCR as no antibodies for these molecules are available that cross-react with the equine counterparts, at least to our knowledge. These molecules are required to trigger and amplify the response of the T-helper cell responses once initial T-cell activation is achieved [6,40] and have been shown to be absent in human MSCs [5,41]. In line with what has been described for human MSCs, no expression of CD86 was detected in equine MSCs. However, we found that our equine MSCs did express CD40 and CD80, with a significantly lower expression for CD80 on UCB-derived MSCs compared with PB-derived MSCs. Differences in expression levels of immunogenic molecules as well as in their release of tolerogenic factors between umbilical cord lining- and BM-derived MSCs have been previously reported in humans, which reinforces the fact that MSCs derived from neonatal sources are preferred from an immunomodulation point of view based on their higher proliferative capacity and lower immunogenicity when compared with MSCs from an adult source [5]. Indeed, it has been demonstrated that fetal-derived human MSCs (1) are less immunogenic, (2) cause less immune activation, (3) get rejected more slowly, (4) were able to suppress lymphocyte proliferation to a significantly greater extent, and (5) are more immunogenically inert, when compared with MSCs derived from adult human sources [5,42-46]. Still, the importance of our findings must be confirmed by evaluating the expression of these molecules on a functional level. On the other hand, MHC-II molecules are essential to initiate the T-cell responses, and since our equine MSCs lack expression of this molecule, it can be anticipated that the presence of CD40 and CD80 might not be that detrimental. Indeed, in a study by Kluyshnenkova and colleagues [41], it was found that transduction of human MSCs (which are also MHC-II-negative) with CD80 failed to elicit a strong proliferative T-cell response. It has to be noted, however, that important differences exist between T-cell subpopulations regarding the requirement for co-stimulatory signals. For example, memory T cells are relatively independent of these signals, and, as such, lower levels of co-stimulation might be sufficient to elicit T-cell responses. Secondly, we also evaluated the expression of several cytokines with known immunomodulatory properties, like TFG-β, HGF, TNF-α, and IDO. TFG-β and HGF, cytokines with immunosuppressive properties, were expressed in MSCs from all three sources. As previously reported, these cytokines function synergistically to suppress T-cell proliferation [41,47]. Moreover, it has been suggested that the basal secretion of TFG-β by equine MSCs may be sufficient to inhibit the T-cell proliferation which is seen *in vitro* after stimulation [3]. As for TNF-α, this is a pro-inflammatory cytokine which mediates inflammation [6]. In mixed lymphocyte reactions in which either allogeneic MSCs or PBMCs were used to elicit T-cell proliferation, reduced levels of TNF-α were detected in response to MSC stimulators when compared with PBMC stimulators [41]. In the study by Yoo and colleagues [2], non-stimulated human MSCs from different sources (that is, BM, adipose tissue, UCB, and UCM) did not secrete TNF-α, with the exception of two isolates from UCB and one isolate from UCM, which is in line with our results. In humans, it is known that IDO is a cytokine which is strongly upregulated in stimulated MSCs and results in the inhibition of T-cell proliferation [2,5]. In the present study, we did not find IDO expression in any MSC sample, but it has to be mentioned that we used non-stimulated MSCs for our qPCR analyses. Still, because it has been described that equine MSCs in the presence of stimulated T cells failed to produce IDO [3], the latter cytokine most likely does not play an important role in defining the immunosuppressive properties of equine MSCs.

## Conclusions

In the present study, a comparative analysis was carried out with equine UCB-, UCM-, and PB-derived MSCs originating from the same horse. Our data strengthen recent findings that inherent differences exist between MSCs from different tissues, as suggested for human MSCs [6]. Combining all the observations in this present study, we propose UCB as the most promising non-invasive alternative source for MSCs and UCM as the least feasible source because of high contamination risks. Moreover, our data indicate that UCB-derived MSCs could be suited for allogeneic use, although their immunogenicity potential needs to be addressed in more detail in future studies.

## Abbreviations

7-AAD: 7-aminoactinomycin D; BM: bone marrow; CDN: cell doubling number; EDTA: ethylenediaminetetraacetic acid; FCS: fetal calf serum; HBSS: Hanks’ balanced salt solution; HGF: hepatocyte growth factor; IDO: indoleamine 2,3-dioxygenase; IFN-γ: interferon-gamma; MHC: major histocompatibility complex; MNC: mononuclear cell; MSC: mesenchymal stromal cell; NCBI: National Center for Biotechnology Information; PB: peripheral blood; PBMC: peripheral blood mononuclear cell; PDT: population doubling time; qPCR: quantitative polymerase chain reaction; RT: room temperature; TGF-β: transforming growth factor-beta; TNF-α: tumor necrosis factor-alpha; UCB: umbilical cord blood; UCM: umbilical cord matrix.

## Competing interests

The authors declare that they have no competing interests.

## Authors’ contributions

CDS was involved in conception and design, sample collection, laboratory analyses, and manuscript writing. KG and KS were involved in the PCR analyses. SP performed the statistical analyses. MH and JG were involved in sample collection and laboratory analyses. EM, AVS, and GVdW were involved in conception and design, data analyses, and manuscript writing. All authors read and approved the final manuscript.
